# 2-Methyl-3-(2-methyl­phen­yl)-4-oxo-3,4-dihydro­quinazolin-8-yl benzoate

**DOI:** 10.1107/S1600536812006253

**Published:** 2012-02-17

**Authors:** Adel S. El-Azab, Alaa A.-M. Abdel-Aziz, Seik Weng Ng, Edward R. T. Tiekink

**Affiliations:** a Department of Pharmaceutical Chemistry, College of Pharmacy, King Saud University, Riyadh 11451, Saudi Arabia; b Department of Organic Chemistry, Faculty of Pharmacy, Al-Azhar University, Cairo 11884, Egypt; c Department of Medicinal Chemistry, Faculty of Pharmacy, University of Mansoura, Mansoura 35516, Egypt; d Department of Chemistry, University of Malaya, 50603 Kuala Lumpur, Malaysia; e Chemistry Department, Faculty of Science, King Abdulaziz University, PO Box 80203 Jeddah, Saudi Arabia

## Abstract

In the title quinazolin-4-one derivative, C_23_H_18_N_2_O_3_, both the benzoate [dihedral angle = 79.99 (6)°] and the 2-tolyl [89.02 (7)°] groups are close to orthogonal to the central fused ring system. Both aryl groups are orientated towards the quinazolin-4-one-bound methyl group. In the crystal, mol­ecules are connected into a three-dimensional architecture by C—H⋯O, C—H⋯N and C—H⋯π inter­actions.

## Related literature

For the pharmacological activity of substituted quinazoline-4(3*H*)-ones, see: El-Azab & El-Tahir (2012 ▶); El-Azab *et al.* (2011 ▶); Al-Omary *et al.* (2010 ▶); Al-Obaid *et al.* (2009 ▶); Aziza *et al.* (1996 ▶). For the synthesis and evaluation of the anti-convulsant activity of the title compound, see: El-Azab *et al.* (2010 ▶). For the structure of the benzoate derivative, see: El-Azab *et al.* (2012 ▶).
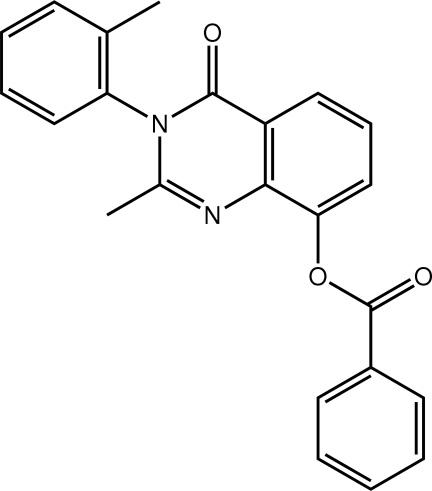


## Experimental

### 

#### Crystal data

C_23_H_18_N_2_O_3_
*M* *_r_* = 370.39
Monoclinic, 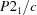

*a* = 20.3847 (4) Å
*b* = 7.4352 (1) Å
*c* = 12.7829 (3) Å
β = 107.489 (2)°
*V* = 1847.87 (6) Å^3^
*Z* = 4
Cu *K*α radiation
μ = 0.72 mm^−1^
*T* = 100 K
0.30 × 0.10 × 0.05 mm

#### Data collection

Agilent SuperNova Dual diffractometer with an Atlas detector
Absorption correction: multi-scan (*CrysAlis PRO*; Agilent, 2011 ▶) *T*_min_ = 0.470, *T*_max_ = 1.000
7377 measured reflections
3780 independent reflections
3432 reflections with *I* > 2σ(*I*)
*R*_int_ = 0.015

#### Refinement

*R*[*F* ^2^ > 2σ(*F* ^2^)] = 0.049
*wR*(*F* ^2^) = 0.132
*S* = 1.05
3780 reflections
255 parameters
H-atom parameters constrained
Δρ_max_ = 0.61 e Å^−3^
Δρ_min_ = −0.28 e Å^−3^

### 

Data collection: *CrysAlis PRO* (Agilent, 2011 ▶); cell refinement: *CrysAlis PRO*; data reduction: *CrysAlis PRO*; program(s) used to solve structure: *SHELXS97* (Sheldrick, 2008 ▶); program(s) used to refine structure: *SHELXL97* (Sheldrick, 2008 ▶); molecular graphics: *ORTEP-3* (Farrugia, 1997 ▶) and *DIAMOND* (Brandenburg, 2006 ▶); software used to prepare material for publication: *publCIF* (Westrip, 2010 ▶).

## Supplementary Material

Crystal structure: contains datablock(s) global, I. DOI: 10.1107/S1600536812006253/hb6635sup1.cif

Structure factors: contains datablock(s) I. DOI: 10.1107/S1600536812006253/hb6635Isup2.hkl

Supplementary material file. DOI: 10.1107/S1600536812006253/hb6635Isup3.cml

Additional supplementary materials:  crystallographic information; 3D view; checkCIF report

## Figures and Tables

**Table 1:** Hydrogen-bond geometry (Å, °) *Cg*1 is the centroid of the C17–C22 benzene ring.

*D*—H⋯*A*	*D*—H	H⋯*A*	*D*⋯*A*	*D*—H⋯*A*
C3—H3⋯N1^i^	0.95	2.58	3.521 (2)	172
C16—H16c⋯O2^ii^	0.98	2.44	3.298 (2)	146
C20—H20⋯O3^iii^	0.95	2.59	3.225 (2)	124
C11—H11⋯*Cg*1^iv^	0.95	2.72	3.5519 (18)	147

Symmetry codes: (i) 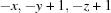; (ii) 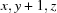; (iii) 
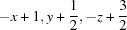
; (iv) 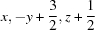.

**Fig. 1.**
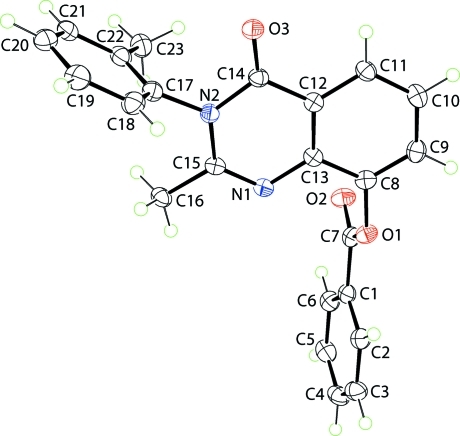
The molecular structure of (I) showing displacement ellipsoids at the 50% probability level.

**Fig. 2.**
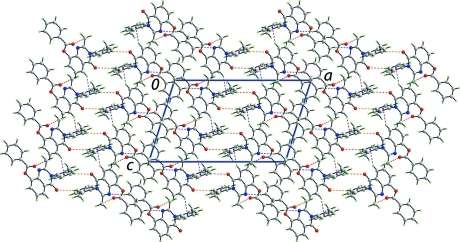
A view in projection down the b axis of the unit-cell contents for (I). The C—H···O, C—H···N and C—H···π interactions are shown as orange, blue and purple dashed lines, respectively.

## supplementary crystallographic information

### Comment

Substituted quinazoline-4(3*H*)-ones are known to display various biological activities (El-Azab & El-Tahir, 2012; El-Azab *et al.*, 2011; El-Azab *et al.*, 2010; Al-Omary *et al.*, 2010; Al-Obaid *et al.*, 2009; Aziza *et al.*, 1996). The title compound, 3,4-dihydro-2-methyl-3-(2-methylphenyl)-4-oxoquinazolin-8-yl benzoate (I), a methaqualone analogue, was recently synthesized and evaluated for its anti-convulsant activity (El-Azab *et al.*, 2010). Herein, the crystal structure determination of (I) is reported.

In (I), Fig. 1, the carboxylate residue is co-planar to the benzene ring to which it is connected as seen in the value of the C2—C1—C7—O1 torsion angle -4.3 (2)°. With respect to the central quinazolin-4-one fused ring system [r.m.s. deviation = 0.035 Å for the 10 atoms], both the benzoate and 2-tolyl groups are orthogonal: the dihedral angles between the central plane and six-membered rings being 79.99 (6) and 89.02 (7)°, respectively. Both aryl substituents are orientated towards the methyl group bound to the quinazolin-4-one system, and the dihedral angle between the two six-membered rings is 64.23 (8)°. The molecular structure resembles closely that of the *p*-tolyl benzoate derivative (El-Azab *et al.*, 2012).

In the crystal packing, C—H···N [involving the quinazolin-N atom], C—H···O [involving both carbonyl-O atoms] and C—H···π [involving the (C17···C22) benzene ring] interactions are formed, Table 1. These lead to a three-dimensional architecture, Fig. 2.

### Experimental

A mixture of 8-hydroxymethaqualone (532 mg, 0.002 *M*) and benzoyl chloride (296 mg, 0.0021 *M*) in 15 ml pyridine was stirred at room temperature for 11 h. The solvent was removed under reduced pressure, and the residue was triturated with water and filtered. The solid obtained was dried and recrystallized from EtOH to yield colourless prisms. *M*.pt.: 438–440. Yield: 94%.

^1^H NMR (500 MHz, CDCl_3_): δ = 8.21 (d, 2H, J = 7.5 Hz), 8.09 (d, 1H, J = 8.0 Hz), 7.82 (d, 1H, J = 8.0 Hz), 7.78 (t, 1H, J = 7.5 Hz), 7.65–7.58 (m, 3H), 7.46–7.38 (m, 4H), 2.04 (s, 3H), 1.99 (s, 3H) p.p.m.. ^13^C NMR (CDCl_3_): δ = 17.3, 24.5, 122.3, 124.7, 127.0, 127.9, 128.2, 128.8, 129.3, 129.5, 129.9, 130.4, 131.6, 134.5, 135.5, 137.1, 140.9, 146.2, 155.5, 160.7, 165.0 p.p.m.. MS (70 eV): m/*z* = 370.

### Refinement

Carbon-bound H-atoms were placed in calculated positions [C—H = 0.95 to 0.98 Å, *U*_iso_(H) = 1.2–1.5*U*_eq_(C)] and were included in the refinement in the riding model approximation. The maximum and minimum residual electron density peaks of 0.61 and 0.28 e Å^-3^, respectively, were located 0.91 Å and 0.53 Å from the C17 and C19 atoms, respectively.

### Crystal data

**Table:**

C_23_H_18_N_2_O_3_	*F*(000) = 776
*M**_r_* = 370.39	*D*_x_ = 1.331 Mg m^−^^3^
Monoclinic, *P*2_1_/*c*	Cu *K*α radiation, λ = 1.5418 Å
Hall symbol: -P 2ybc	Cell parameters from 4141 reflections
*a* = 20.3847 (4) Å	θ = 3.6–76.0°
*b* = 7.4352 (1) Å	µ = 0.72 mm^−^^1^
*c* = 12.7829 (3) Å	*T* = 100 K
β = 107.489 (2)°	Prism, colourless
*V* = 1847.87 (6) Å^3^	0.30 × 0.10 × 0.05 mm
*Z* = 4	

### Data collection

**Table:**

Agilent SuperNova Dual diffractometer with an Atlas detector	3780 independent reflections
Radiation source: SuperNova (Cu) X-ray Source	3432 reflections with *I* > 2σ(*I*)
Mirror monochromator	*R*_int_ = 0.015
Detector resolution: 10.4041 pixels mm^-1^	θ_max_ = 76.1°, θ_min_ = 4.6°
ω scan	*h* = −22→25
Absorption correction: multi-scan (*CrysAlis PRO*; Agilent, 2011)	*k* = −9→6
*T*_min_ = 0.470, *T*_max_ = 1.000	*l* = −15→15
7377 measured reflections	

### Refinement

**Table:**

Refinement on *F*^2^	Primary atom site location: structure-invariant direct methods
Least-squares matrix: full	Secondary atom site location: difference Fourier map
*R*[*F*^2^ > 2σ(*F*^2^)] = 0.049	Hydrogen site location: inferred from neighbouring sites
*wR*(*F*^2^) = 0.132	H-atom parameters constrained
*S* = 1.05	*w* = 1/[σ^2^(*F*_o_^2^) + (0.0621*P*)^2^ + 1.4481*P*] where *P* = (*F*_o_^2^ + 2*F*_c_^2^)/3
3780 reflections	(Δ/σ)_max_ = 0.001
255 parameters	Δρ_max_ = 0.61 e Å^−^^3^
0 restraints	Δρ_min_ = −0.28 e Å^−^^3^

### Special details

**Table:**

Geometry. All e.s.d.'s (except the e.s.d. in the dihedral angle between two l.s. planes) are estimated using the full covariance matrix. The cell e.s.d.'s are taken into account individually in the estimation of e.s.d.'s in distances, angles and torsion angles; correlations between e.s.d.'s in cell parameters are only used when they are defined by crystal symmetry. An approximate (isotropic) treatment of cell e.s.d.'s is used for estimating e.s.d.'s involving l.s. planes.
Refinement. Refinement of *F*^2^ against ALL reflections. The weighted *R*-factor *wR* and goodness of fit *S* are based on *F*^2^, conventional *R*-factors *R* are based on *F*, with *F* set to zero for negative *F*^2^. The threshold expression of *F*^2^ > σ(*F*^2^) is used only for calculating *R*-factors(gt) *etc*. and is not relevant to the choice of reflections for refinement. *R*-factors based on *F*^2^ are statistically about twice as large as those based on *F*, and *R*- factors based on ALL data will be even larger.

### Fractional atomic coordinates and isotropic or equivalent isotropic displacement parameters (Å^2^)

**Table:**

	*x*	*y*	*z*	*U*_iso_*/*U*_eq_	
O1	0.12153 (6)	0.28357 (15)	0.64163 (9)	0.0228 (3)	
O2	0.17292 (6)	0.08881 (17)	0.55602 (10)	0.0287 (3)	
O3	0.39006 (6)	0.65559 (18)	0.84640 (10)	0.0284 (3)	
N1	0.21618 (7)	0.52042 (18)	0.60240 (11)	0.0197 (3)	
N2	0.31826 (7)	0.69110 (19)	0.67202 (11)	0.0209 (3)	
C1	0.05574 (8)	0.1737 (2)	0.46895 (13)	0.0220 (3)	
C2	−0.00084 (9)	0.2669 (2)	0.48010 (14)	0.0248 (4)	
H2	0.0028	0.3330	0.5452	0.030*	
C3	−0.06272 (9)	0.2636 (2)	0.39634 (16)	0.0295 (4)	
H3	−0.1014	0.3278	0.4038	0.035*	
C4	−0.06782 (10)	0.1661 (2)	0.30169 (16)	0.0313 (4)	
H4	−0.1101	0.1640	0.2443	0.038*	
C5	−0.01162 (10)	0.0715 (2)	0.29002 (15)	0.0301 (4)	
H5	−0.0156	0.0047	0.2251	0.036*	
C6	0.05038 (9)	0.0750 (2)	0.37355 (14)	0.0258 (4)	
H6	0.0890	0.0107	0.3660	0.031*	
C7	0.12288 (8)	0.1733 (2)	0.55681 (13)	0.0212 (3)	
C8	0.18435 (8)	0.3093 (2)	0.72251 (13)	0.0212 (3)	
C9	0.19716 (9)	0.2213 (2)	0.82114 (14)	0.0248 (4)	
H9	0.1646	0.1378	0.8323	0.030*	
C10	0.25825 (9)	0.2546 (2)	0.90526 (14)	0.0254 (4)	
H10	0.2674	0.1922	0.9730	0.030*	
C11	0.30502 (9)	0.3773 (2)	0.89015 (13)	0.0225 (3)	
H11	0.3466	0.3995	0.9470	0.027*	
C12	0.29094 (8)	0.4695 (2)	0.79007 (13)	0.0195 (3)	
C13	0.23106 (8)	0.4352 (2)	0.70379 (12)	0.0187 (3)	
C14	0.33798 (8)	0.6088 (2)	0.77586 (13)	0.0205 (3)	
C15	0.25920 (8)	0.6410 (2)	0.59004 (12)	0.0192 (3)	
C16	0.24555 (9)	0.7342 (2)	0.48214 (13)	0.0238 (3)	
H16A	0.2042	0.6836	0.4300	0.036*	
H16B	0.2849	0.7174	0.4540	0.036*	
H16C	0.2387	0.8629	0.4916	0.036*	
C17	0.36264 (9)	0.8369 (2)	0.65716 (13)	0.0250 (4)	
C18	0.35070 (9)	1.0096 (2)	0.69125 (15)	0.0276 (4)	
H18	0.3126	1.0318	0.7177	0.033*	
C19	0.39532 (9)	1.1478 (2)	0.68583 (15)	0.0303 (4)	
H19	0.3875	1.2663	0.7071	0.036*	
C20	0.45170 (9)	1.1116 (3)	0.64898 (14)	0.0290 (4)	
H20	0.4829	1.2055	0.6469	0.035*	
C21	0.46277 (9)	0.9408 (2)	0.61541 (14)	0.0269 (4)	
H21	0.5012	0.9189	0.5898	0.032*	
C22	0.41779 (9)	0.7985 (2)	0.61865 (13)	0.0250 (4)	
C23	0.43021 (10)	0.6146 (2)	0.58317 (15)	0.0297 (4)	
H23A	0.4362	0.5307	0.6444	0.045*	
H23B	0.4718	0.6148	0.5599	0.045*	
H23C	0.3908	0.5770	0.5218	0.045*	

### Atomic displacement parameters (Å^2^)

**Table:**

	*U*^11^	*U*^22^	*U*^33^	*U*^12^	*U*^13^	*U*^23^
O1	0.0196 (5)	0.0213 (6)	0.0261 (6)	−0.0029 (4)	0.0049 (5)	−0.0053 (4)
O2	0.0236 (6)	0.0286 (6)	0.0320 (6)	0.0019 (5)	0.0052 (5)	−0.0065 (5)
O3	0.0232 (6)	0.0370 (7)	0.0205 (6)	−0.0089 (5)	−0.0003 (5)	0.0019 (5)
N1	0.0201 (6)	0.0176 (6)	0.0194 (6)	0.0010 (5)	0.0031 (5)	−0.0019 (5)
N2	0.0207 (7)	0.0233 (7)	0.0172 (6)	−0.0032 (5)	0.0034 (5)	0.0002 (5)
C1	0.0230 (8)	0.0166 (7)	0.0250 (8)	−0.0042 (6)	0.0053 (6)	0.0010 (6)
C2	0.0256 (8)	0.0181 (7)	0.0299 (9)	−0.0026 (6)	0.0070 (7)	−0.0002 (6)
C3	0.0249 (9)	0.0220 (8)	0.0385 (10)	0.0004 (7)	0.0049 (7)	0.0034 (7)
C4	0.0288 (9)	0.0246 (8)	0.0322 (9)	−0.0049 (7)	−0.0033 (7)	0.0055 (7)
C5	0.0370 (10)	0.0244 (8)	0.0249 (8)	−0.0061 (7)	0.0031 (7)	−0.0011 (7)
C6	0.0278 (8)	0.0218 (8)	0.0274 (8)	−0.0024 (7)	0.0075 (7)	−0.0015 (6)
C7	0.0239 (8)	0.0154 (7)	0.0251 (8)	−0.0030 (6)	0.0083 (7)	−0.0013 (6)
C8	0.0207 (8)	0.0183 (7)	0.0234 (8)	−0.0002 (6)	0.0046 (6)	−0.0042 (6)
C9	0.0287 (9)	0.0187 (8)	0.0285 (8)	−0.0036 (6)	0.0110 (7)	−0.0005 (6)
C10	0.0334 (9)	0.0211 (8)	0.0218 (8)	0.0001 (7)	0.0084 (7)	0.0016 (6)
C11	0.0254 (8)	0.0219 (8)	0.0186 (7)	0.0004 (6)	0.0043 (6)	−0.0005 (6)
C12	0.0212 (7)	0.0180 (7)	0.0190 (7)	0.0007 (6)	0.0058 (6)	−0.0019 (6)
C13	0.0213 (7)	0.0164 (7)	0.0186 (7)	0.0018 (6)	0.0060 (6)	−0.0021 (6)
C14	0.0199 (7)	0.0230 (8)	0.0177 (7)	0.0004 (6)	0.0043 (6)	−0.0011 (6)
C15	0.0194 (7)	0.0188 (7)	0.0182 (7)	0.0018 (6)	0.0036 (6)	−0.0025 (6)
C16	0.0253 (8)	0.0233 (8)	0.0192 (7)	−0.0009 (6)	0.0012 (6)	0.0012 (6)
C17	0.0241 (8)	0.0292 (9)	0.0190 (7)	−0.0042 (7)	0.0023 (6)	0.0024 (6)
C18	0.0259 (8)	0.0253 (8)	0.0301 (9)	−0.0018 (7)	0.0062 (7)	−0.0002 (7)
C19	0.0323 (9)	0.0246 (9)	0.0317 (9)	−0.0009 (7)	0.0061 (7)	−0.0031 (7)
C20	0.0259 (8)	0.0296 (9)	0.0276 (8)	−0.0036 (7)	0.0018 (7)	0.0009 (7)
C21	0.0258 (8)	0.0282 (9)	0.0227 (8)	−0.0013 (7)	0.0013 (6)	0.0062 (7)
C22	0.0261 (8)	0.0251 (8)	0.0207 (8)	0.0007 (7)	0.0027 (6)	0.0022 (6)
C23	0.0333 (9)	0.0270 (9)	0.0286 (9)	−0.0011 (7)	0.0090 (7)	0.0018 (7)

### Geometric parameters (Å, º)

**Table:**

O1—C7	1.3661 (19)	C10—C11	1.375 (2)
O1—C8	1.3962 (19)	C10—H10	0.9500
O2—C7	1.201 (2)	C11—C12	1.403 (2)
O3—C14	1.219 (2)	C11—H11	0.9500
N1—C15	1.296 (2)	C12—C13	1.401 (2)
N1—C13	1.392 (2)	C12—C14	1.459 (2)
N2—C15	1.3887 (19)	C15—C16	1.493 (2)
N2—C14	1.406 (2)	C16—H16A	0.9800
N2—C17	1.461 (2)	C16—H16B	0.9800
C1—C2	1.389 (2)	C16—H16C	0.9800
C1—C6	1.399 (2)	C17—C22	1.386 (3)
C1—C7	1.487 (2)	C17—C18	1.400 (3)
C2—C3	1.388 (2)	C18—C19	1.387 (3)
C2—H2	0.9500	C18—H18	0.9500
C3—C4	1.387 (3)	C19—C20	1.393 (3)
C3—H3	0.9500	C19—H19	0.9500
C4—C5	1.390 (3)	C20—C21	1.381 (3)
C4—H4	0.9500	C20—H20	0.9500
C5—C6	1.388 (2)	C21—C22	1.409 (2)
C5—H5	0.9500	C21—H21	0.9500
C6—H6	0.9500	C22—C23	1.486 (2)
C8—C9	1.374 (2)	C23—H23A	0.9800
C8—C13	1.406 (2)	C23—H23B	0.9800
C9—C10	1.401 (2)	C23—H23C	0.9800
C9—H9	0.9500		
			
C7—O1—C8	115.93 (12)	C11—C12—C14	119.88 (14)
C15—N1—C13	117.52 (13)	N1—C13—C12	123.02 (14)
C15—N2—C14	122.18 (14)	N1—C13—C8	119.49 (14)
C15—N2—C17	122.11 (13)	C12—C13—C8	117.48 (14)
C14—N2—C17	115.68 (13)	O3—C14—N2	120.83 (15)
C2—C1—C6	120.09 (15)	O3—C14—C12	124.70 (15)
C2—C1—C7	121.84 (15)	N2—C14—C12	114.47 (13)
C6—C1—C7	118.07 (15)	N1—C15—N2	123.95 (14)
C3—C2—C1	120.16 (16)	N1—C15—C16	119.25 (14)
C3—C2—H2	119.9	N2—C15—C16	116.80 (14)
C1—C2—H2	119.9	C15—C16—H16A	109.5
C4—C3—C2	119.63 (17)	C15—C16—H16B	109.5
C4—C3—H3	120.2	H16A—C16—H16B	109.5
C2—C3—H3	120.2	C15—C16—H16C	109.5
C3—C4—C5	120.66 (16)	H16A—C16—H16C	109.5
C3—C4—H4	119.7	H16B—C16—H16C	109.5
C5—C4—H4	119.7	C22—C17—C18	122.36 (16)
C6—C5—C4	119.82 (17)	C22—C17—N2	119.55 (16)
C6—C5—H5	120.1	C18—C17—N2	117.88 (15)
C4—C5—H5	120.1	C19—C18—C17	119.04 (17)
C5—C6—C1	119.65 (17)	C19—C18—H18	120.5
C5—C6—H6	120.2	C17—C18—H18	120.5
C1—C6—H6	120.2	C18—C19—C20	119.58 (17)
O2—C7—O1	122.72 (15)	C18—C19—H19	120.2
O2—C7—C1	125.87 (15)	C20—C19—H19	120.2
O1—C7—C1	111.41 (13)	C21—C20—C19	120.75 (17)
C9—C8—O1	119.55 (15)	C21—C20—H20	119.6
C9—C8—C13	121.44 (15)	C19—C20—H20	119.6
O1—C8—C13	118.85 (14)	C20—C21—C22	120.87 (17)
C8—C9—C10	119.99 (15)	C20—C21—H21	119.6
C8—C9—H9	120.0	C22—C21—H21	119.6
C10—C9—H9	120.0	C17—C22—C21	117.38 (16)
C11—C10—C9	120.24 (15)	C17—C22—C23	121.95 (16)
C11—C10—H10	119.9	C21—C22—C23	120.66 (16)
C9—C10—H10	119.9	C22—C23—H23A	109.5
C10—C11—C12	119.46 (15)	C22—C23—H23B	109.5
C10—C11—H11	120.3	H23A—C23—H23B	109.5
C12—C11—H11	120.3	C22—C23—H23C	109.5
C13—C12—C11	121.35 (15)	H23A—C23—H23C	109.5
C13—C12—C14	118.73 (14)	H23B—C23—H23C	109.5
			
C6—C1—C2—C3	−0.5 (2)	O1—C8—C13—C12	174.56 (13)
C7—C1—C2—C3	−179.84 (15)	C15—N2—C14—O3	−179.91 (15)
C1—C2—C3—C4	0.3 (3)	C17—N2—C14—O3	−1.9 (2)
C2—C3—C4—C5	0.1 (3)	C15—N2—C14—C12	−0.8 (2)
C3—C4—C5—C6	−0.3 (3)	C17—N2—C14—C12	177.21 (14)
C4—C5—C6—C1	0.1 (3)	C13—C12—C14—O3	176.86 (16)
C2—C1—C6—C5	0.3 (3)	C11—C12—C14—O3	−1.0 (3)
C7—C1—C6—C5	179.68 (15)	C13—C12—C14—N2	−2.2 (2)
C8—O1—C7—O2	7.2 (2)	C11—C12—C14—N2	179.96 (14)
C8—O1—C7—C1	−172.79 (13)	C13—N1—C15—N2	−0.9 (2)
C2—C1—C7—O2	175.74 (16)	C13—N1—C15—C16	179.36 (14)
C6—C1—C7—O2	−3.6 (3)	C14—N2—C15—N1	2.5 (2)
C2—C1—C7—O1	−4.3 (2)	C17—N2—C15—N1	−175.36 (15)
C6—C1—C7—O1	176.36 (14)	C14—N2—C15—C16	−177.75 (14)
C7—O1—C8—C9	−103.54 (17)	C17—N2—C15—C16	4.4 (2)
C7—O1—C8—C13	81.03 (17)	C15—N2—C17—C22	−92.04 (19)
O1—C8—C9—C10	−176.08 (14)	C14—N2—C17—C22	89.96 (18)
C13—C8—C9—C10	−0.8 (3)	C15—N2—C17—C18	93.06 (19)
C8—C9—C10—C11	1.0 (3)	C14—N2—C17—C18	−84.94 (19)
C9—C10—C11—C12	0.3 (3)	C22—C17—C18—C19	0.4 (3)
C10—C11—C12—C13	−1.9 (2)	N2—C17—C18—C19	175.12 (15)
C10—C11—C12—C14	175.82 (15)	C17—C18—C19—C20	−1.4 (3)
C15—N1—C13—C12	−2.4 (2)	C18—C19—C20—C21	1.5 (3)
C15—N1—C13—C8	177.21 (14)	C19—C20—C21—C22	−0.7 (3)
C11—C12—C13—N1	−178.26 (14)	C18—C17—C22—C21	0.5 (2)
C14—C12—C13—N1	4.0 (2)	N2—C17—C22—C21	−174.21 (14)
C11—C12—C13—C8	2.1 (2)	C18—C17—C22—C23	179.70 (16)
C14—C12—C13—C8	−175.64 (14)	N2—C17—C22—C23	5.0 (2)
C9—C8—C13—N1	179.61 (15)	C20—C21—C22—C17	−0.3 (2)
O1—C8—C13—N1	−5.0 (2)	C20—C21—C22—C23	−179.57 (16)
C9—C8—C13—C12	−0.8 (2)		

### Hydrogen-bond geometry (Å, º)

Cg1 is the centroid of the C17–C22 benzene ring.

**Table:**

*D*—H···*A*	*D*—H	H···*A*	*D*···*A*	*D*—H···*A*
C3—H3···N1^i^	0.95	2.58	3.521 (2)	172
C16—H16c···O2^ii^	0.98	2.44	3.298 (2)	146
C20—H20···O3^iii^	0.95	2.59	3.225 (2)	124
C11—H11···*Cg*1^iv^	0.95	2.72	3.5519 (18)	147

Symmetry codes: (i) −*x*, −*y*+1, −*z*+1; (ii) *x*, *y*+1, *z*; (iii) −*x*+1, *y*+1/2, −*z*+3/2; (iv) *x*, −*y*+3/2, *z*+1/2.
